# Heterogeneity of the *Stearoyl-CoA desaturase-1* (*SCD1*) Gene and Metabolic Risk Factors in the EPIC-Potsdam Study

**DOI:** 10.1371/journal.pone.0048338

**Published:** 2012-11-06

**Authors:** Maria Arregui, Brian Buijsse, Norbert Stefan, Dolores Corella, Eva Fisher, Romina di Giuseppe, Oscar Coltell, Sven Knüppel, Krasimira Aleksandrova, Hans-Georg Joost, Heiner Boeing, Cornelia Weikert

**Affiliations:** 1 Department of Epidemiology, German Institute of Human Nutrition (DIfE) Potsdam-Rehbruecke, Nuthetal, Germany; 2 Department of Internal Medicine, Division of Endocrinology, Diabetology, Nephrology, Vascular Disease and Clinical Chemistry, Member of the Deutsches Zentrum für Diabetesforschung (DZD), University of Tübingen, Tübingen, Germany; 3 Department of Preventive Medicine and CIBER Fisiopatología de la Obesidad y Nutrición, School of Medicine, University of Valencia, Valencia, Spain; 4 Administrative Office of the Commission on Genetic Testing, Robert Koch-Institute, Berlin, Germany; 5 Department of Computing Languages and Systems, Universitat Jaume I, Castellón, Spain; 6 Department of Pharmacology, German Institute of Human Nutrition (DIfE) Potsdam-Rehbruecke, Nuthetal, Germany; 7 Institute for Social Medicine, Epidemiology, and Health Economics, Charité University Medical Center, Berlin, Germany; Kunming Institute of Zoology, Chinese Academy of Sciences, China

## Abstract

**Background:**

Stearoyl-CoA desaturase-1 (SCD1) is an enzyme involved in lipid metabolism. In mice and humans its activity has been associated with traits of the metabolic syndrome, but also with the prevention of saturated fatty acids accumulation and subsequent inflammation, whereas for liver fat content inconsistent results have been reported. Thus, variants of the gene encoding SCD1 (*SCD1*) could potentially modify metabolic risk factors, but few human studies have addressed this question.

**Methods:**

In a sample of 2157 middle-aged men and women randomly drawn from the Potsdam cohort of the European Prospective Investigation into Cancer and Nutrition, we investigated the impact of 7 *SCD1* tagging-single nucleotide polymorphisms (rs1502593, rs522951, rs11190480, rs3071, rs3793767, rs10883463 and rs508384) and 5 inferred haplotypes with frequency >5% describing 90.9% of the genotype combinations in our population, on triglycerides, body mass index (BMI), waist circumference (WC), glycated haemoglobin (HbA1c), high-sensitivity C-reactive protein (hs-CRP), gamma-glutamyltransferase (GGT), alanine aminotransferase (ALT) and fetuin-A.

**Results:**

No significant associations between any of the SNPs or haplotypes and BMI, WC, fetuin-A and hs-CRP were observed. Associations of rs10883463 with triglycerides, GGT and HbA1c as well as of rs11190480 with ALT activity, were weak and became non-significant after multiple-testing correction. Also associations of the haplotype harbouring the minor allele of rs1502593 with HbA1c levels, the haplotype harbouring the minor alleles of rs11190480 and rs508384 with activity of ALT, and the haplotype harbouring the minor alleles of rs522951, rs10883463 and rs508384 with triglyceride and HbA1C levels and GGT activities did not withstand multiple-testing correction.

**Conclusion:**

These findings suggest that there are no associations between common variants of *SCD1* or its inferred haplotypes and the investigated metabolic risk factors. However, given the results from animal models, heterogeneity of human *SCD1* warrants further investigation, in particular with regard to rare variants.

## Introduction

The human stearoyl-CoA desaturase-1 (*SCD1*) gene maps to chromosome 10q24.31, has 6 exons and is remarkably expressed in adipose tissue and the liver. It encodes the endoplasmatic reticulum enzyme SCD1, which catalyses the conversion of the saturated fatty acids (SFAs) palmitic and stearic, into the monounsaturated fatty acids (MUFAs) palmitoleic and oleic respectively [1]. These MUFAs are the major components of triglycerides, likely due to its production within the environs of the enzyme diacylglycerol acyltransferase (DGAT) [1]. Accordingly, it has been suggested that the increased activity of SCD1 in the liver, could result in an excess assembly and accumulation of triglycerides and subsequent development of hepatic steatosis [2]. Further, the overflow of triglycerides could also be incorporated into very low density lipoprotein (VLDL) particles and transported to adipose tissue and other sites, contributing to the development of obesity [3]. Both these conditions have been associated with insulin resistance [4]. Additionally, SCD1 deficiency has been associated with the reduced expression of fatty acid synthesis genes [5] and the up-regulation of genes involved in fatty acid β-oxidation [5], [6]. In fact mice with a natural or a targeted deletion of the *SCD1* gene, have shown to be protected against hypertriglyceridemia [7], hepatic steatosis [8]–[10], obesity [3], [5], [8], [11], [12] and insulin resistance [11], [13], [14]. Conversely, there is also evidence that by channelling SFA, into triglyceride pools, increased SCD1 activity may prevent from lipoapoptosis [15], steatohepatitis [2] and inflammation [6], [15]–[17]. Therefore, SCD1 seams to convey both, positive and negative roles in the development of metabolic risk factors of cardiovascular diseases.

Despite the strong scientific interest in SCD1, most of the existing knowledge on its function comes from mice models [18]. Some human studies have provided indirect evidence of the role of its activity (approximated as fatty acids product-to-precursor ratios measured in serum, plasma, erythrocytes or adipose tissue), and have proposed that its elevation might be associated with harmful effects such as elevated plasma triglycerides levels [19], [20], liver fat [21]–[23], obesity [19], [24], diabetes [25], high-sensitivity C-reactive protein (hs-CRP) levels [26] and even with cardiovascular mortality [27]. Conversely the scarce human studies investigating the tissue-specific activity and expression of *SCD1*, suggest that elevated SCD1 activity may protect from liver fat accumulation [28], [29]. Further, the impact of *SCD1* heterogeneity on metabolic risk factors, has so far only been investigated in four human studies with focus on diabetes and obesity [30], [31], metabolic syndrome (MetS) [32] or inflammation [33]. Thus, a case control study in men and women from the United Kingdom, found no associations between 6 *SCD1* single nucleotide polymorphisms (SNPs) or its inferred haplotypes and diabetes, body mass index (BMI) or waist-to-hip ratio [30]; a cross-sectional study in Swedish elderly men reported that 4 out of 8 *SCD1* tagging SNPs (tag-SNPs) related to decreased BMI and waist circumference (WC), and increased insulin sensitivity [31]; the haplotype consisting of the rare alleles of these SNPs was also associated with decreased WC; a cross-sectional study in Costa Rican middle-aged men and women reported that 1 out of 7 *SCD1* tag-SNPs was associated with an increased prevalence of MetS, and among women, also with elevated systolic blood pressure and fasting blood glucose levels [32]. Also 2 haplotypes carrying the minor allele of this SNP were associated with elevated prevalence of MetS; finally a cross-sectional study in European and Asian young adults found 1 out of 10 tag-SNPs to be associated with CRP levels [33].

In the Potsdam cohort of the European Prospective Investigation into Cancer and Nutrition (EPIC-Potsdam), we investigated the impact of common genetic variation in *SCD1*, captured by means of 7 tag-SNPs and their inferred haplotypes, on the modulation of 8 metabolic risk factors related to the activity of SCD1. The investigated traits were plasma triglyceride levels, traits related to obesity (BMI and waist circumference (WC)), glucose metabolism (glycated haemoglobin (HbA1c)) and chronic inflammation (hs-CRP), and, for the first time, crude estimates of the presence of liver fat (the liver enzymes gamma-glutamyltransferase (GGT) and alanine amino transferase (ALT) [34], [35]), and fetuin-A, a biomarker which has been associated with fat accumulation in the liver [36]–[40] as well as with insulin resistance, type 2 diabetes and cardiovascular events [40]–[42].

## Methods

### Ethics Statement

Written informed consent was obtained from all study participants, and approval was given by the Ethics Committee of the Medical Association of the State of Brandenburg, Germany.

### Study Population

EPIC-Potsdam comprises 27548 individuals (10904 men and 16644 women) from the general population of the Potsdam area in Germany. Men were mainly aged 40–65 and women 35–65 years old at recruitment, which took place between 1994 and 1998 [43]. The baseline examination included a personal interview and a questionnaire on sociodemographic and lifestyle characteristics and prevalent diseases as well as anthropometric measurements [44]. The associations of 7 *SCD1* tag-SNPs and their inferred haplotypes with anthropometric and metabolic markers were investigated in a random sample of 2500 individuals (subcohort) drawn from the participants in the total cohort who had provided blood samples at baseline, following a cross-sectional design. After exclusion of individuals with missing covariates or genotype data, the final study population comprised 2157 participants. Fasting was not required at the time of blood draw, however, 615 participants were in fasting state for at least 8 h.

### Laboratory Analyses

From all the study participants a 30 mL sample of venous blood was collected, fractionated into serum, plasma, buffy coat and erythrocytes, and stored in liquid nitrogen until the time of analysis. Plasma levels of triglycerides, HbA1c, GGT, ALT, fetuin-A, hs-CRP, total cholesterol and high density lipoprotein (HDL)-cholesterol were determined with the automatic ADVIA 1650 analyser (Siemens Medical Solutions, Erlangen, Germany) at the Department of Internal Medicine of the University of Tübingen, Germany, in 2007.

### SNP Selection and Genotyping

Seven *SCD1* tag-SNPs (ordered according to chromosomic location: rs1502593, rs522951, rs11190480, rs3071, rs3793767, rs10883463 and rs508384) were identified in the HapMap 22/phaseII CEU population data (Utah residents with ancestry from northern and western Europe) [45] using stringent criteria (minor allele frequency (MAF) >0.05 and pairwise r^2^≥0.8) by means of the Tagger software [46] implemented in the version 4.2 of Haploview [47]. The tagged region comprised the coding region of *SCD1* as well as a 4.1 Kb upstream (promoter) and 4.3 Kb downstream (3′ untranslated) region of the gene. Six SNPs were located in intronic sites and one in the 3′ untranslated region (rs508384). Genotyping of whole genome amplified DNA samples was performed with a 7900HT Sequence Detection System with TaqMan assays (Applied Biosystems, Foster City, CA, USA) at the Max Delbrück Centre for Molecular Medicine, Berlin, Germany, in 2009. The average genotyping success rate in the 7 SNPs was >98%.

### Statistical Analyses

Normality of variables was tested by estimating their skewness and kurtosis, by comparing their means and median values and by plotting their distributions in histograms. To better reach normality of their distributions, triglycerides, GGT, ALT and hs-CRP were natural log-transformed and HbA1c inverse-transformed, and were used like that in all analyses. Hardy–Weinberg equilibrium (HWE) of the SNPs was tested using the χ^2^ test. Linkage disequilibrium between SNPs was assessed with the r^2^ measure using Haploview 4.2 [47]. Each SNP was coded as 0, 1 and 2 according to the number of minor alleles a participant carried. Analysis of covariance considering the additive, dominant and recessive genetic models was used to assess the associations between the SNPs (independent variables) and triglycerides, BMI, WC, HbA1c, GGT, ALT, fetuin-A and hs-CRP (dependent variables). Haplotypes were constructed to test whether multiple genetic variants of *SCD1* or a possible unobserved risk variant captured by the haplotypes, modulated the investigated traits. Haplotype frequencies were estimated based on the observed unphased genotypes by the expectation-maximization algorithm [48]. The effects of a particular haplotype load (0, 1 or 2 copies) were tested also by means of a regression-based analysis (ANCOVA) as suggested by Zaykin et al. [49]. Analyses were restricted to participants with a probability of 49–50% to carry one copy of the haplotype, or 100% probability to carry either none or two copies. Only haplotypes with frequencies >5% were considered. The additive, dominant and recessive models were examined. Analyses for triglycerides were performed only in participants who were fasting at the time of blood draw. Data are reported as means and standard errors, geometric means and 95% confidence intervals (CI) or inverse and 95% CI as appropriate. Regression coefficients and standard errors (SE) were also estimated. All analyses were adjusted for age and sex. Further (mutual) adjustment for known cardiovascular risk factors including smoking status (never smoker, former smoker, current smoker <20 cigarettes per day, current smoker ≥20 cigarettes per day), sports activity (<2 h/wk versus ≥2 h/wk), educational attainment (vocational school or less, technical school, university), BMI (continuous), WC (continuous), alcohol consumption (men: = 0 g/d, >0 to 12 g/d, >12 to 24 g/d; >24 g/d; women: = 0 g/d, >0 to 6 g/d, >6 to 12 g/d; >12 g/d), prevalent diabetes, prevalent hypertension, total cholesterol, HDL cholesterol and hs-CRP) were also explored for the tag-SNPs. Effect modification by sex was evaluated by modeling the cross product term sex times genotype or haplotype, along with main effects (in the age-adjusted general linear model). All statistical analyses were performed using SAS Enterprise Guide 4.3 (SAS Institute Inc., Cary, NC, USA).

Power calculations were performed with Quanto [50] considering an additive model, a desirable power of 80%, a two-sided α of 0.05, the means and standard deviations of the traits in the subcohort (non-normal variables were transformed to normality) and the genotype frequencies of the least (rs10883463, MAF = 8%) and most (rs522951, MAF = 46%) common tag-SNPs. The detectable differences in our study ranged between 0.1 and 0.2 SD for BMI, WC, GGT, ALT, fetuin-A, hs-CRP and HbA1c and between 0.2 and 0.3 SD for triglycerides. Conservative Bonferroni correction for multiple comparisons was performed (P _Bonferroni_ = α/(n individual hypothesis tested) = 0.05/((7 SNPs +5 haplotypes)×3 genetic models per SNP×8 traits investigated). The corrected significance threshold was P _Bonferroni_ = 0.0002.

## Results

### Characteristics of the Study Population

Demographic, lifestyle, clinical, biochemical and genetic characteristics of the study population are given in **Table 1** **,** both for the subcohort and separately for men and women. Men (38%) were older than women due to sampling strategy. After adjusting for age, they also showed to smoke and drink more and to take more often antidiabetic medication. Further their BMI, WC and their activities of GGT and ALT were higher. They were more likely to be higher educated and also had lower hs-CRP levels than women. The genotype frequency of all *SCD1* tag-SNPs followed HWE (P>0.05), their allele frequencies were comparable to those observed in HapMap 22/phaseII CEU population data [45] and did not differ among sexes. **Table S1** presents further information regarding genotype and allelic frequencies of the tag-SNPs across the EPIC-Potsdam subcohort and separately for men and women.

**Table 1:** Baseline characteristics of the EPIC-Potsdam subcohort and separately for men and women.

	Subcohort	Men	Women	P value a
Characteristic	(N = 2157)	(N = 819; 38%)	(N = 1338; 62% )	
**Age** (years)	50.3±9.0	52.4 (51.8–53.0)	49.0 (48.5–49.4)	<0.0001
**Educational attainment** (%):				
Vocational school or less	37.4	30.8	41.5	<0.0001
Technical school	24.9	15.9	30.4	<0.0001
University degree	37.7	53.4	28.1	<0.0001
**Physical activity** (%):				
<2 hours/week	75.9	74.7	76.7	0.3
≥2 hours/week	24.1	25.3	23.3	0.3
**Smoking status** (%):				
Current ≥20 cigarettes/d	6.3	11.3	3.2	<0.0001
Current <20 cigarettes/d	14.9	16.7	13.8	0.07
Former ≤5 years	7.5	9.5	6.3	0.006
Former >5 years	24.4	35.3	17.7	<0.0001
Never	46.9	27.2	59.0	<0.0001
**Alcohol intake** (%):				
Men: = 0 g/d; women: = 0 g/d	0.05	0.1	0.0	0.3
Men: >0–12 g/d; women: >0–6 g/d	47.2	36.4	53.9	<0.0001
Men: >12–24 g/d; women: >6–12 g/d	24.7	26.1	23.9	0.3
Men: >24 g/d; women: >12 g/d	28.0	37.5	22.2	<0.0001
**Use of medication** (%):				
Antidiabetic	2.6	3.5	2.0	0.03
Antihypertensive	19.3	19.6	19.2	0.8
Lipid lowering medication	5.1	5.7	4.7	0.3
**Body mass index** (kg/m^2^)	26.1±4.3	26.6 (26.3–26.8)	25.8 (25.6–26.1)	0.0002
**Waist circumference** (cm)	85.8±12.9	93.4 (92.6–94.1)	81.2 (80.6–81.7)	<0.0001
**Triglycerides** b (mg/dL)	90.2 (64.9–126.5)	109.3 (102.1–117.0)	82.1 (77.6–86.7)	<0.0001
**Gamma-glutamyltransferase** (U/L)	16.8 (11.0–30.8)	28.3 (26.8–29.9)	14.4 (13.8–15.0)	<0.0001
**Glutamic-pyruvate transaminase** (U/L)	18.7 (14.3–26.4)	25.9(25.1–26.7)	16.8 (16.4–17.3)	<0.0001
**Fetuin-A** c (mg/dL)	0.25±0.06	0.25 (0.24–0.25)	0.25 0.25–0.26)	0.03
**Glycated haemoglobin** (%)	6.4 (6.1–6.8)	6.5 (6.4–6.6)	6.4 (6.4–6.5)	0.002
**Hs-C-reactive protein** (mg/L)	0.8 (0.2–2.1)	0.6 (0.6–0.7)	0.8 (0.6–0.7)	<0.0001
**Minor allele frequency** d (%):				
rs1502593 (C>T)	44	45	43	0.2
rs522951 (G>C)	46	46	47	0.6
rs11190480 (A>G)	9	9	9	0.6
rs3071 (T>G)	35	35	34	0.8
rs3793767 (T>C)	38	36	39	0.2
rs10883463 (T>C)	8	8	8	0.7
rs508384 (C>A)	17	17	17	1.0

**Subcohort**: Mean ± SD, %, or median (25^th^ percentile; 75^th^ percentile), all such values. **Men and women**: mean and 95% confidence interval (CI) or %. Results obtained using analysis of covariance, all variables other than age are adjusted for age.

a P value for the difference between men and women.

b based on the 615 participants fasting at blood draw.

c based on 2077 participants due to missing biomarker data.

d Alleles given in brackets (most >less frequent allele).

Linkage disequilibrium values (r^2^) between each pair of tag-SNPs ranged from 0 to 0.66. Five haplotypes inferred from the genotyped SNPs, with frequency >5% described 90.9% of the genotype combinations in our population: A-B-A-A-B-A-A (34.1%); B-A-A-B-A-A-A (29.8%), B-A-A-A-A-A-A (11.0%), A-A-B-A-A-A-B (8.6%) and A-B-A-A-A-B-B (7.4%) (Haplotypes are composed of variants rs1502593 (C>T), rs522951 (G>C), rs11190480 (A>G), rs3071 (T>G), rs3793767 (T>C), rs10883463 (T>C), rs508384 (C>A) in that order; A indicates common allele, B indicates rare allele).

### Associations between *SCD1* Tag-SNPs and Inferred Haplotypes and the Investigated Traits

No significant effect modifications by sex were found for any of the tag-SNPs or inferred haplotypes on the investigated traits, thus results are presented combined for men and women.

**Table 2** shows results for age- and sex-adjusted mean values of the 8 investigated metabolic traits for each of the 7 *SCD1* tag-SNPs. No significant associations were found between rs1502593, rs522951, rs3071, rs3793767 or rs508384 and any of the investigated traits. Also no significant associations were found between any of the investigated SNPs and fetuin-A, BMI, WC or hs-CRP. However, carriers of the rs11190480 rare allele, presented slightly lower activities of ALT in a dominant fashion (19.91 vs. 21.07 U/L, P = 0.03). Carriers of the rs10883463 rare allele, showed higher triglycerides (160.97 vs. 94.02 mg/dL, P = 0.03) and lower HbA1c levels (6.10 vs. 6.49%, P = 0.03) in a recessive fashion, and slightly higher activities of GGT (22.22 vs. 19.88 U/L, P = 0.02) in a dominant fashion. Results were weak in precision, as shown by the wide confidence intervals, and after applying the Bonferroni correction for the multiple hypothesis tested (P _Bonferroni_ = 0.0002), none of them remained significant. After further (mutual) adjustment of the statistical models for known cardiovascular risk factors, results remained essentially similar (**table S2**). Also they were not modified by adjustment for fasting status or exclusion of participants taking lipid-lowering or antidiabetic medication.

**Table 2:** Age- and sex-adjusted association analyses between the 7 *SCD1* tag-SNPs and the 8 investigated metabolic traits: the EPIC-Potsdam Study.

	Triglyceridesa, b (mg/dL)	BMI c(kg/m^2^)	WC c(cm)	HbA1c d (%)	GGT b (U/L)	ALT b (U/L)	Fetuin-A c, e(mg/dL)	hs-CRP b(mg/L)
**rs1502593 (n)**								
0 (679)	96.45 (89.23–104.26)	26.22±0.16	87.47±0.41	6.48 (6.43–6.53)	20.13 (18.97–21.35)	20.52 (19.81–21.25)	0.25±0.002	0.73 (0.66–0.81)
1 (1072)	92.49 (86.98–98.34)	26.25±0.13	87.36±0.33	6.50 (6.46–6.55)	20.24 (19.31–21.21)	20.85 (20.28–21.44)	0.25±0.002	0.72 (0.67–0.79)
2 (406)	96.46 (87.67–106.14)	26.00±0.21	86.63±0.53	6.45 (6.39–6.52)	20.27 (18.79–21.87)	21.52 (20.57–22.50)	0.26±0.003	0.73 (0.63–0.83)
P_add_	0.89	0.47	0.25	0.63	0.87	0.11	0.19	0.88
P_dom_	0.53	0.83	0.53	0.80	0.87	0.24	0.75	0.86
P_rec_	0.63	0.30	0.18	0.25	0.93	0.14	0.06	0.96
**rs522951 (n)**								
0 (607)	94.06 (86.85–101.88)	26.03±0.17	86.79±0.44	6.47 (6.41–6.52)	19.93 (18.73–21.21)	20.96 (20.20–21.75)	0.25±0.002	0.72 (0.64–0.80)
1 (1095)	95.22 (89.54–101.26)	26.23±0.13	87.26±0.32	6.50 (6.46–6.54)	20.46 (19.53–21.43)	20.88 (20.31–21.46)	0.25±0.002	0.73 (0.67–0.79)
2 (455)	93.32 (85.14–102.30)	26.31±0.20	87.87±0.50	6.49 (6.43–6.55)	19.98 (18.59–21.47)	20.73 (19.87–21.64)	0.25±0.003	0.73 (0.64–0.83)
P_add_	0.93	0.28	0.10	0.50	0.90	0.70	0.46	0.82
P_dom_	0.90	0.27	0.20	0.35	0.60	0.78	0.20	0.80
P_rec_	0.77	0.52	0.17	0.90	0.72	0.73	0.87	0.91
**rs11190480 (n)**								
0 (1787)	93.97 (89.60–98.56)	26.23±0.10	87.38±0.26	6.49 (6.46–5.52)	20.30 (19.57–21.06)	21.08 (20.62–21.54)	0.25±0.001	0.73 (0.68–0.78)
1 (357)	96.89 (87.11–107.77)	26.05±0.22	86.66±0.57	6.46 (6.39–6.53)	19.93 (18.39–21.61)	19.85 (18.92–20.82)	0.25±0.003	0.72 (0.63–0.83)
2 (13)	114.86 (39.83–331.24)	25.60±1.16	86.71±2.95	6.66 (6.29–7.06)	15.46 (10.14–23.57)	21.67 (16.88–27.81)	0.24±0.015	0.59 (0.28–1.25)
P_add_	0.56	0.39	0.25	0.63	0.41	0.05	0.35	0.78
P_dom_	0.58	0.42	0.24	0.49	0.54	0.03	0.36	0.85
P_rec_	0.72	0.61	0.85	0.38	0.21	0.77	0.73	0.59
**rs3071 (n)**								
0 (944)	95.09 (89.07–101.53)	26.36±0.14	87.65±0.35	6.47 (6.43–6.52)	20.64 (19.63–21.70)	20.75 (20.15–21.38)	0.25±0.002	0.77(0.71–0.84)
1 (936)	94.84 (88.81–101.29)	25.96±0.14	86.79±0.35	6.50 (6.45–6.54)	19.76 (18.79–20.78)	20.86 (20.25–21.49)	0.25±0.002	0.68(0.63–0.75)
2 (277)	91.32 (81.28–102.61)	26.42±0.25	87.49±0.64	6.52 (6.44–6.60)	20.30 (18.53–22.25)	21.30 (20.17–22.48)	0.25±0.003	0.73(0.63–0.86)
P_add_	0.62	0.47	0.36	0.28	0.46	0.46	0.92	0.22
P_dom_	0.79	0.10	0.13	0.32	0.27	0.62	0.86	0.08
P_rec_	0.54	0.34	0.69	0.46	0.92	0.43	0.64	0.90
**rs3793767 (n)**								
0 (845)	95.94 (89.64–102.68)	26.17±0.15	87.21±0.37	6.47 (6.42–6.51)	20.50 (19.45–21.61)	21.05 (20.41–21.72)	0.25±0.002	0.71 (0.65–0.78)
1 (998)	94.63 (88.81–100.83)	26.15±0.13	87.15±0.34	6.51 (6.47–6.55)	20.40 (19.43–21.41)	20.83 (20.23–21.44)	0.25±0.002	0.74 (0.68–0.81)
2 (314)	89.68 (79.88–100.67)	26.41±0.24	87.74±0.61	6.48 (6.41–6.56)	18.86 (17.30–20.57)	20.51 (19.49–21.59)	0.25±0.003	0.73 (0.62–0.84)
P_add_	0.37	0.50	0.57	0.50	0.17	0.38	0.97	0.65
P_dom_	0.56	0.81	0.86	0.26	0.49	0.48	0.50	0.50
P_rec_	0.34	0.33	0.38	0.81	0.09	0.47	0.32	0.97
**rs10883463 (n)**								
0 (1840)	94.64 (90.34–99.15)	26.18±0.10	87.21±0.25	6.49 (6.46–6.52)	19.88 (19.17–20.61)	20.78 (20.34–21.23)	0.25±0.001	0.73 (0.68–0.78)
1 (304)	89.89 (79.74–101.33)	26.24±0.24	87.39±0.61	6.49 (6.41–6.56)	22.43 (20.56–24.48)	21.35 (20.27–22.48)	0.25±0.003	0.72 (0.62–0.85)
2 (13)	160.99 (100.50–257.90)	27.49±1.16	90.77±2.95	6.10 (5.80–6.44)	17.85 (11.72–27.19)	22.48(17.52–28.85)	0.23±0.017	0.61 (0.29–1.28)
P_add_	0.76	0.50	0.48	0.36	0.04	0.27	0.85	0.81
P_dom_	0.80	0.64	0.63	0.62	0.02	0.30	0.99	0.88
P_rec_	0.03	0.26	0.23	0.03	0.56	0.56	0.32	0.63
**rs508384 (n)**								
0 (1489)	93.94 (89.21–98.93)	26.18±0.11	87.27±0.28	6.50 (6.46–6.53)	20.01 (19.22–20.83)	21.01 (20.51–21.51)	0.25±0.002	0.73 (0.68–0.78)
1 (610)	93.61 (86.21–101.65)	26.24±0.17	87.16±0.43	6.47 (6.42–6.53)	20.66 (19.42–21.98)	20.55 (19.81–21.31)	0.25±0.002	0.74 (0.66–0.82)
2 (58)	119.44 (92.43–154.35)	26.18±0.55	87.90±1.40	6.44 (6.27–6.62)	20.67 (16.92–25.25)	20.85 (18.52–23.48)	0.25±0.008	0.64 (0.45–0.91)
P_add_	0.36	0.81	0.96	0.36	0.40	0.39	0.27	0.86
P_dom_	0.69	0.78	0.92	0.39	0.38	0.33	0.31	0.95
P_rec_	0.07	0.98	0.64	0.58	0.82	0.99	0.49	0.46

Each SNP is coded as 0, 1 and 2 according to the number of minor alleles a participant carries.

a based on the 615 participants fasting at blood draw.

b geometric means and (95% CI);

c means and standard error;

d inverse and (95% CI),

e based on 2077 participants due to missing biomarker data. All the reported significance levels are nominal P values and are not adjusted for multiple comparisons.

**P_add_**, P for trend or P for the additive model; **P_dom_**, P value for the dominant model. **P_rec_**, P value for the recessive model.

**Table 3** shows results for age- and sex-adjusted mean values of the investigated metabolic traits for each of the 5 *SCD1* haplotypes. No associations between the two most common haplotypes and the investigated variables became apparent. Homozygotes for haplotype B-A-A-A-A-A-A harbouring the minor allele of the SNP rs1502593 presented lower HbA1c levels (6.03 vs. 6.49%, P = 0.003). Carriers of haplotype A-A-B-A-A-A-B harbouring the minor alleles of the SNPs rs11190480 and rs508384 exhibited slightly lower activities of ALT in a dominant fashion (19.85 vs. 21.08 U/L, P = 0.02). Carriers of haplotype A-B-A-A-A-B-B harbouring the minor alleles of the SNPs rs522951, rs10883463 and rs508384 showed higher triglyceride values 179.77 vs. 94.08 mg/dL and lower HbA1C levels (6.09 vs. 6.49%, P = 0.03) in a recessive fashion, and higher GGT activities (22.24 vs. 19.93 U/L, P = 0.03) in a dominant fashion. However, after correction for multiple testing, also none of these results remained significant. **Table S3** summarizes all of these results in the form of age- and sex-adjusted regression coefficients.

**Table 3:** Age- and sex-adjusted association analyses between the 5 *SCD1* inferred haplotypes with frequency >5% and the 8 investigated metabolic traits in the EPIC-Potsdam study.

Haplotype (%)	N (%)	Triglycerides a, b (mg/dL)	BMI c (kg/m^2^)	WC c (cm)	HbA1c d (%)	GGT b (U/L)	ALT b (U/L)	Fetuin-A c, e (mg/dL)	hs-CRP b (mg/L)
**A-B-A-A-B-A-A** (34.1)									
0 copies	919 (43.13)	95.76 (89.70–102.24)	26.18±0.14	87.20±0.35	6.47 (6.43–6.52)	20.59 (19.58–21.66)	21.07 (20.45–21.71)	0.25±0.002	0.71 (0.65–0.77)
1 copy	962 (45.14)	93.61 (87.77–99.85)	26.18±0.14	87.23±0.35	6.50 (6.46–6.55)	20.17 (19.20–21.20)	20.83 (20.23–21.45)	0.25±0.002	0.74 (0.68–0.81)
2 copies	250 (11.73)	91.55 (80.82–103.70)	26.37±0.27	87.84±0.68	6.48 (6.40–6.56)	18.88 (17.14–20.81)	20.43 (19.29–21.64)	0.25±0.004	0.72 (0.61–0.85)
P_add_		0.49	0.63	0.51	0.58	0.15	0.34	0.70	0.59
P_dom_		0.54	0.83	0.74	0.37	0.31	0.44	0.54	0.44
P_rec_		0.62	0.51	0.39	0.82	0.14	0.42	0.89	0.94
**B-A-A-B-A-A-A** (29.8)									
0 copies	1041 (48.94)	94.50 (88.75–100.64)	26.29±0.13	87.58±0.33	6.48 (6.44–6.52)	20.73 (19.76–21.74)	20.73 (20.15–21.33)	0.25±0.002	0.76 (0.70–0.82)
1 copy	885 (41.61)	95.12 (88.91–101.77)	26.07±0.14	86.98±0.36	6.51 (6.46–6.55)	19.64 (18.65–20.69)	20.91 (20.27–21.56)	0.25±0.002	0.69 (0.63–0.76)
2 copies	201 (9.45)	86.77 (75.63–99.56)	26.15±0.30	86.59±0.75	6.46 (6.37–6.55)	20.02 (17.98–22.30)	21.35 (20.03–22.75)	0.25±0.004	0.71 (0.59–0.86)
P_add_		0.46	0.35	0.13	0.75	0.23	0.42	0.93	0.25
P_dom_		0.80	0.25	0.14	0.44	0.14	0.54	0.82	0.16
P_rec_		0.23	0.88	0.36	0.55	0.87	0.46	0.56	0.86
**B-A-A-A-A-A-A** (11.0)									
0 copies	1213 (73.34)	92.97 (87.36–98.93)	26.22±0.13	87.20±0.32	6.48 (6.45–6.52)	19.99 (19.11–20.91)	20.76 (20.22–21.32)	0.25±0.002	0.72 (0.66–0.77)
1 copy	425 (25.7)	97.72 (88.58–107.81)	26.23±0.21	87.69±0.52	6.49 (6.43–6.55)	21.45 (19.92–23.10)	21.03 (20.14–21.97)	0.25±0.003	0.77 (0.68–0.88)
2 copies	16 (0.97)	96.50 (63.43–146.81)	25.35±1.07	84.24±2.69	6.03 (5.76–6.33)	23.32 (15.94–34.12)	24.69 (19.74–30.88)	0.27±0.015	0.91 (0.47–1.79)
P_add_		0.42	0.79	0.74	0.33	0.08	0.31	0.36	0.26
P_dom_		0.40	0.92	0.55	0.69	0.09	0.46	0.53	0.30
P_rec_		0.91	0.41	0.25	0.003	0.48	0.14	0.11	0.51
**A-A-B-A-A-A-B** (8.6)									
0 copies	1795 (83.29)	93.99 (89.62–98.57)	26.23±0.10	87.38±0.26	6.49 (6.46–6.52)	20.30 (19.56–21.06)	21.08 (20.63–21.55)	0.25±0.001	0.73 (0.68–0.78)
1 copy	348 (16.15)	96.83 (87.00–107.76)	26.07±0.23	86.71±0.57	6.46 (6.39–6.53)	20.01 (18.44–21.72)	19.79 (18.85–20.77)	0.25±0.003	0.73 (0.63–0.85)
2 copies	12 (0.56)	114.87 (39.83–331.27)	26.10±1.21	87.90±3.07	6.68 (6.30–7.11)	17.22 (11.10–26.72)	21.96 (16.94–28.48)	0.25±0.017	0.57 (0.26–1.23)
P_add_		0.57	0.54	0.35	0.70	0.59	0.04	0.32	0.88
P_dom_		0.60	0.52	0.31	0.55	0.67	0.02	0.29	0.97
P_rec_		0.72	0.94	0.84	0.33	0.47	0.70	0.87	0.53
**A-B-A-A-A-B-B** (7.4)									
0 copies	1855 (86.6)	94.51 (90.23–99.00)	26.17±0.10	87.21±0.25	6.49 (6.46–6.52)	19.93 (19.22–20.66)	20.80 (20.36–21.25)	0.25±0.001	0.73 (0.68–0.77)
1 copy	275 (12.84)	90.86 (80.18–102.97)	26.30±0.25	87.54±0.64	6.51 (6.43–6.59)	22.43 (20.47–24.59)	21.40 (20.27–22.60)	0.25±0.004	0.71 (0.61–0.84)
2 copies	12 (0.56)	179.79 (106.09–304.69)	27.90±1.20	92.07±3.07	6.09 (5.78–6.45)	18.23 (11.76–28.26)	22.96 (17.70–29.78)	0.23±0.017	0.66 (0.30–1.42)
P_add_		0.62	0.32	0.30	0.63	0.05	0.25	0.75	0.76
P_dom_		0.96	0.45	0.44	0.97	0.03	0.28	0.91	0.79
P_rec_		0.02	0.16	0.12	0.03	0.64	0.48	0.30	0.80

Haplotypes are composed of variants rs1502593 (C>T), rs522951 (G>C), rs11190480 (A>G), rs3071 (T>G), rs3793767 (T>C), rs10883463 (T>C), rs508384 (C>A) in that order. A indicates common allele, B indicates rare allele.

a based on the 615 participants fasting at blood draw.

b geometric means and (95% CI);

c means and standard error;

d inverse and (95% CI);

e based on 2077 participants due to missing biomarker data. All the reported significance levels are nominal P values and are not adjusted for multiple comparisons.

**P_add_**: P for trend or P for the additive model; **P_dom_**: P value for the dominant model. **P_rec_**: P value for the recessive model.

## Discussion

In the present study of a middle-aged sample of German men and women, we evaluated the impact of 7 *SCD1* tag-SNPs and 5 inferred haplotypes on MetS related traits on suggested crude estimates of the presence of liver fat and on inflammation. Our study is, so far, the largest performed in a European population, and also the first to report association results between *SCD1* genetic variants and liver parameters. We hypothesized that any functional variant affecting the activity of SCD1 would possibly result in the modulation of one or more of the traits. At most, we found some associations weak in magnitude, precision, and statistical significance, which after conservative Bonferroni-correction for the number of traits, SNPs and haplotypes tested, did not remain significant, thus being suggestive of chance findings.

Four previous studies in humans have investigated the association of *SCD1* polymorphisms with different metabolic traits [30]–[33]. In a UK case-control study of 608 cases and 600 controls, Liew et al. [30] reported upon the association of 6 *SCD1* SNPs with type 2 diabetes, BMI and waist-to-hip ratio. Three of these SNPs were in common or highly linked to SNPs of our study: rs670213 (a good proxy for rs522951, r^2^ = 0.87 according to HapMap data for Caucasians of European origin [45], rs3071 and rs11598233 (a perfect proxy for rs3793767, r^2^ = 1 [45]). Consistent with our results, they also reported no significant associations. Warensjö et al. [31] investigated associations of 8 *SCD1* tag-SNPs with obesity and insulin sensitivity in 1143 Swedish elderly men. Five of these SNPs, were in common or highly linked to SNPs of our study, rs3870747 (linked with rs1119040, r^2^ = 0.94 [45]), rs3071, rs3793767, rs10883463 and rs508384. In line with our results, they found a tendency of rs10883463 carriers towards increased insulin sensitivity, and no significant associations for rs3870747 and rs3793767. In contrast, they reported lower WC in carriers of rs10883463, lower WC and higher insulin sensitivity in homozygotes for rs508384 rare allele and lower insulin sensitivity in heterozygotes for rs3071. This last association was also inconsistent with the results from Liew et al. [30]. Compared with our study population, the study of Warensjö et al. [31] was smaller and included only elderly men. However, we found no significant sex interaction for any of the SNPs with any of the studied phenotypes, and further all our association analyses were adjusted for age, what makes the overall evidence for an association of *SCD1* genetic variability with WC and insulin sensitivity less consistent. Recently, Gong et al. [32] analysed the association of 7 *SCD1* tag-SNPs with MetS prevalence in 2152 Costa Rican adult men and women. Only one SNP, rs1502593, was found to be associated with MetS. This SNP was also analysed in our population. In line with our results, they did not observe significant associations between rs1502593 and triglyceride levels or WC. They did point out a borderline association of rs1502593 with elevated fasting blood glucose levels among women. In our population we found no significant associations for this SNP. Homozygotes for the haplotype harbouring its minor allele showed lower HbA1c levels, measure of the average plasma glucose levels over prolonged periods of time, but this association did not withhold after multiple-testing correction. We cannot discard the possibility that the different ethnic origin may explain the different findings. Finally, Stryjecki et al. [33] examined the relationships between 10 *SCD1* tag-SNPs and CRP levels in 279 European and 249 Asian young adults. Only one SNP located 9 Kb upstream *SCD1,* and thus not included in our study, was associated with CRP levels, and only among females of both groups.

Limitations of our study should be mentioned. It is possible that, due to sample size limitations we were not able to detect minor contributions of the alleles. This limitation was stronger in the case of the association analyses for triglycerides levels, as fasting data was available only for about one third of the population. Thus while some of our results could represent a replication for certain relationships inspected in the four association studies that precede ours [30]–[33], to evaluate the outcome of our work, further studies in independent cohorts analysing the same SNPs, or those in perfect linkage disequilibrium, are necessary [51]. Also, we cannot exclude the possibility that a rare causal variant exists within the typed region but was not picked up by the chosen markers.

In summary, our findings suggest that common variants of *SCD1* do not modulate the investigated metabolic factors in this European population. However, given its biological relevance, the still scarce number of studies available and the inconsistency of their results, genetic heterogeneity of human *SCD1* in relation to impaired metabolism rewards further investigation in independent study populations, in particular with regard to rare variants of *SCD1*.

## Supporting Information

Table S1 Genotype and allelic frequencies of the *SCD1* tag-SNPs across the EPIC-Potsdam subcohort and separately for men and women.
(PDF)

Table S2 Association analysis between the 7 *SCD1* tag-SNPs and the 8 investigated metabolic traits in the EPIC-Potsdam Study, (mutually) adjusted for known cardiovascular risk factors.
(PDF)

Table S3 β regression coefficients for the age- and sex-adjusted association analysis between the *SCD1* tag-SNPs and inferred haplotypes and the 8 investigated metabolic traits in the EPIC-Potsdam study.
(PDF)

## References

[1] Paton CM, Ntambi JM (2009) Biochemical and physiological function of stearoyl-CoA desaturase. Am J Physiol Endocrinol Metab 297: E28–37. 19066317 10.1152/ajpendo.90897.2008 PMC2711665

[2] Li ZZ, Berk M, McIntyre TM, Feldstein AE (2009) Hepatic lipid partitioning and liver damage in nonalcoholic fatty liver disease: role of stearoyl-CoA desaturase. J Biol Chem 284: 5637–5644. 19119140 10.1074/jbc.M807616200 PMC2645822

[3] Cohen P, Miyazaki M, Socci ND, Hagge-Greenberg A, Liedtke W, et al (2002) Role for stearoyl-CoA desaturase-1 in leptin-mediated weight loss. Science 297: 240–243. 12114623 10.1126/science.1071527

[4] Petersen KF, Shulman GI (2006) Etiology of insulin resistance. Am J Med 119: S10–16. 16563942 10.1016/j.amjmed.2006.01.009 PMC2995525

[5] Ntambi JM, Miyazaki M, Stoehr JP, Lan H, Kendziorski CM, et al (2002) Loss of stearoyl-CoA desaturase-1 function protects mice against adiposity. Proc Natl Acad Sci U S A 99: 11482–11486. 12177411 10.1073/pnas.132384699 PMC123282

[6] MacDonald ML, van Eck M, Hildebrand RB, Wong BW, Bissada N, et al (2009) Despite antiatherogenic metabolic characteristics, SCD1-deficient mice have increased inflammation and atherosclerosis. Arterioscler Thromb Vasc Biol 29: 341–347. 19095997 10.1161/ATVBAHA.108.181099 PMC2873333

[7] Miyazaki M, Kim YC, Gray-Keller MP, Attie AD, Ntambi JM (2000) The biosynthesis of hepatic cholesterol esters and triglycerides is impaired in mice with a disruption of the gene for stearoyl-CoA desaturase 1. J Biol Chem 275: 30132–30138. 10899171 10.1074/jbc.M005488200

[8] Miyazaki M, Dobrzyn A, Sampath H, Lee SH, Man WC, et al (2004) Reduced adiposity and liver steatosis by stearoyl-CoA desaturase deficiency are independent of peroxisome proliferator-activated receptor-alpha. J Biol Chem 279: 35017–35024. 15180999 10.1074/jbc.M405327200

[9] Miyazaki M, Dobrzyn A, Man WC, Chu K, Sampath H, et al (2004) Stearoyl-CoA desaturase 1 gene expression is necessary for fructose-mediated induction of lipogenic gene expression by sterol regulatory element-binding protein-1c-dependent and -independent mechanisms. J Biol Chem 279: 25164–25171. 15066988 10.1074/jbc.M402781200

[10] Miyazaki M, Flowers MT, Sampath H, Chu K, Otzelberger C, et al (2007) Hepatic stearoyl-CoA desaturase-1 deficiency protects mice from carbohydrate-induced adiposity and hepatic steatosis. Cell Metab 6: 484–496. 18054317 10.1016/j.cmet.2007.10.014

[11] MacDonald ML, Singaraja RR, Bissada N, Ruddle P, Watts R, et al (2008) Absence of stearoyl-CoA desaturase-1 ameliorates features of the metabolic syndrome in LDLR-deficient mice. J Lipid Res 49: 217–229. 17960025 10.1194/jlr.M700478-JLR200 PMC5017869

[12] Sampath H, Flowers MT, Liu X, Paton CM, Sullivan R, et al (2009) Skin-specific deletion of stearoyl-CoA desaturase-1 alters skin lipid composition and protects mice from high fat diet-induced obesity. J Biol Chem 284: 19961–19973. 19429677 10.1074/jbc.M109.014225 PMC2740422

[13] Gutierrez-Juarez R, Pocai A, Mulas C, Ono H, Bhanot S, et al (2006) Critical role of stearoyl-CoA desaturase-1 (SCD1) in the onset of diet-induced hepatic insulin resistance. J Clin Invest 116: 1686–1695. 16741579 10.1172/JCI26991 PMC1464900

[14] Flowers JB, Rabaglia ME, Schueler KL, Flowers MT, Lan H, et al (2007) Loss of stearoyl-CoA desaturase-1 improves insulin sensitivity in lean mice but worsens diabetes in leptin-deficient obese mice. Diabetes 56: 1228–1239. 17369521 10.2337/db06-1142

[15] Listenberger LL, Han X, Lewis SE, Cases S, Farese RV Jr, et al (2003) Triglyceride accumulation protects against fatty acid-induced lipotoxicity. Proc Natl Acad Sci U S A 100: 3077–3082. 12629214 10.1073/pnas.0630588100 PMC152249

[16] Liu X, Strable MS, Ntambi JM (2011) Stearoyl CoA desaturase 1: role in cellular inflammation and stress. Adv Nutr 2: 15–22. 22211186 10.3945/an.110.000125 PMC3042787

[17] Brown JM, Chung S, Sawyer JK, Degirolamo C, Alger HM, et al (2008) Inhibition of stearoyl-coenzyme A desaturase 1 dissociates insulin resistance and obesity from atherosclerosis. Circulation 118: 1467–1475. 18794388 10.1161/CIRCULATIONAHA.108.793182 PMC2716169

[18] Sampath H, Ntambi JM (2011) The role of stearoyl-CoA desaturase in obesity, insulin resistance, and inflammation. Ann N Y Acad Sci 1243: 47–53. 22211892 10.1111/j.1749-6632.2011.06303.x

[19] Zhou YE, Egeland GM, Meltzer SJ, Kubow S (2009) The association of desaturase 9 and plasma fatty acid composition with insulin resistance-associated factors in female adolescents. Metabolism 58: 158–166. 19154947 10.1016/j.metabol.2008.09.008

[20] Petersson H, Basu S, Cederholm T, Riserus U (2008) Serum fatty acid composition and indices of stearoyl-CoA desaturase activity are associated with systemic inflammation: longitudinal analyses in middle-aged men. Br J Nutr 99: 1186–1189. 18062827 10.1017/S0007114507871674

[21] Petersson H, Arnlov J, Zethelius B, Riserus U (2010) Serum fatty acid composition and insulin resistance are independently associated with liver fat markers in elderly men. Diabetes Res Clin Pract 87: 379–384. 20022128 10.1016/j.diabres.2009.11.019

[22] Kotronen A, Seppanen-Laakso T, Westerbacka J, Kiviluoto T, Arola J, et al (2009) Hepatic stearoyl-CoA desaturase (SCD)-1 activity and diacylglycerol but not ceramide concentrations are increased in the nonalcoholic human fatty liver. Diabetes 58: 203–208. 18952834 10.2337/db08-1074 PMC2606873

[23] Tomita K, Teratani T, Yokoyama H, Suzuki T, Irie R, et al (2011) Plasma free myristic acid proportion is a predictor of nonalcoholic steatohepatitis. Dig Dis Sci 56: 3045–3052. 21516322 10.1007/s10620-011-1712-0

[24] Gong J, Campos H, McGarvey S, Wu Z, Goldberg R, et al (2011) Adipose tissue palmitoleic acid and obesity in humans: does it behave as a lipokine? Am J Clin Nutr 93: 186–191. 21084651 10.3945/ajcn.110.006502 PMC3001604

[25] Kroger J, Zietemann V, Enzenbach C, Weikert C, Jansen EH, et al (2011) Erythrocyte membrane phospholipid fatty acids, desaturase activity, and dietary fatty acids in relation to risk of type 2 diabetes in the European Prospective Investigation into Cancer and Nutrition (EPIC)-Potsdam Study. Am J Clin Nutr 93: 127–142. 20980488 10.3945/ajcn.110.005447

[26] Petersson H, Lind L, Hulthe J, Elmgren A, Cederholm T, et al (2009) Relationships between serum fatty acid composition and multiple markers of inflammation and endothelial function in an elderly population. Atherosclerosis 203: 298–303. 18687433 10.1016/j.atherosclerosis.2008.06.020

[27] Warensjö E, Sundstrom J, Vessby B, Cederholm T, Riserus U (2008) Markers of dietary fat quality and fatty acid desaturation as predictors of total and cardiovascular mortality: a population-based prospective study. Am J Clin Nutr 88: 203–209. 18614742 10.1093/ajcn/88.1.203

[28] Stefan N, Peter A, Cegan A, Staiger H, Machann J, et al (2008) Low hepatic stearoyl-CoA desaturase 1 activity is associated with fatty liver and insulin resistance in obese humans. Diabetologia 51: 648–656. 18286258 10.1007/s00125-008-0938-7

[29] Peter A, Cegan A, Wagner S, Elcnerova M, Konigsrainer A, et al (2011) Relationships between hepatic stearoyl-CoA desaturase-1 activity and mRNA expression with liver fat content in humans. Am J Physiol Endocrinol Metab 300: E321–326. 21045174 10.1152/ajpendo.00306.2010

[30] Liew CF, Groves CJ, Wiltshire S, Zeggini E, Frayling TM, et al (2004) Analysis of the contribution to type 2 diabetes susceptibility of sequence variation in the gene encoding stearoyl-CoA desaturase, a key regulator of lipid and carbohydrate metabolism. Diabetologia 47: 2168–2175. 15662557 10.1007/s00125-004-1575-4

[31] Warensjö E, Ingelsson E, Lundmark P, Lannfelt L, Syvanen AC, et al (2007) Polymorphisms in the SCD1 gene: associations with body fat distribution and insulin sensitivity. Obesity (Silver Spring) 15: 1732–1740. 17636091 10.1038/oby.2007.206

[32] Gong J, Campos H, McGarvey S, Wu Z, Goldberg R, et al (2011) Genetic variation in stearoyl-CoA desaturase 1 is associated with metabolic syndrome prevalence in Costa Rican adults. J Nutr 141: 2211–2218. 22049297 10.3945/jn.111.143503 PMC3223878

[33] Stryjecki C, Roke K, Clarke S, Nielsen D, Badawi A, et al.. (2011) Enzymatic activity and genetic variation in SCD1 modulate the relationship between fatty acids and inflammation. Mol Genet Metab. 10.1016/j.ymgme.2011.12.003 22209225

[34] Stefan N, Kantartzis K, Haring HU (2008) Causes and metabolic consequences of Fatty liver. Endocr Rev 29: 939–960. 18723451 10.1210/er.2008-0009

[35] Schindhelm RK, Diamant M, Dekker JM, Tushuizen ME, Teerlink T, et al (2006) Alanine aminotransferase as a marker of non-alcoholic fatty liver disease in relation to type 2 diabetes mellitus and cardiovascular disease. Diabetes Metab Res Rev 22: 437–443. 16832839 10.1002/dmrr.666

[36] Yilmaz Y, Yonal O, Kurt R, Ari F, Oral AY, et al (2010) Serum fetuin A/alpha2HS-glycoprotein levels in patients with non-alcoholic fatty liver disease: relation with liver fibrosis. Ann Clin Biochem 47: 549–553. 20926473 10.1258/acb.2010.010169

[37] Haukeland JW, Dahl TB, Yndestad A, Gladhaug IP, Loberg EM, et al (2012) Fetuin A in nonalcoholic fatty liver disease: in vivo and in vitro studies. Eur J Endocrinol 166: 503–510. 22170794 10.1530/EJE-11-0864

[38] Mussig K, Staiger H, Machicao F, Machann J, Hennige AM, et al (2009) AHSG gene variation is not associated with regional body fat distribution–a magnetic resonance study. Exp Clin Endocrinol Diabetes 117: 432–437. 19358088 10.1055/s-0028-1103299

[39] Reinehr T, Roth CL (2008) Fetuin-A and its relation to metabolic syndrome and fatty liver disease in obese children before and after weight loss. J Clin Endocrinol Metab 93: 4479–4485. 18728159 10.1210/jc.2008-1505

[40] Stefan N, Hennige AM, Staiger H, Machann J, Schick F, et al (2006) Alpha2-Heremans-Schmid glycoprotein/fetuin-A is associated with insulin resistance and fat accumulation in the liver in humans. Diabetes Care 29: 853–857. 16567827 10.2337/diacare.29.04.06.dc05-1938

[41] Stefan N, Fritsche A, Weikert C, Boeing H, Joost HG, et al (2008) Plasma fetuin-A levels and the risk of type 2 diabetes. Diabetes 57: 2762–2767. 18633113 10.2337/db08-0538 PMC2551687

[42] Weikert C, Stefan N, Schulze MB, Pischon T, Berger K, et al (2008) Plasma fetuin-a levels and the risk of myocardial infarction and ischemic stroke. Circulation 118: 2555–2562. 19029462 10.1161/CIRCULATIONAHA.108.814418

[43] Boeing H, Korfmann A, Bergmann MM (1999) Recruitment procedures of EPIC-Germany. European Investigation into Cancer and Nutrition. Ann Nutr Metab 43: 205–215. 10592369 10.1159/000012787

[44] Kroke A, Bergmann MM, Lotze G, Jeckel A, Klipstein-Grobusch K, et al (1999) Measures of quality control in the German component of the EPIC study. European Prospective Investigation into Cancer and Nutrition. Ann Nutr Metab 43: 216–224. 10592370 10.1159/000012788

[45] Consortium IH (2005) A haplotype map of the human genome. Nature 437: 1299–1320. 16255080 10.1038/nature04226 PMC1880871

[46] de Bakker PI, Yelensky R, Pe’er I, Gabriel SB, Daly MJ, et al (2005) Efficiency and power in genetic association studies. Nat Genet 37: 1217–1223. 16244653 10.1038/ng1669

[47] Barrett JC, Fry B, Maller J, Daly MJ (2005) Haploview: analysis and visualization of LD and haplotype maps. Bioinformatics 21: 263–265. 15297300 10.1093/bioinformatics/bth457

[48] Excoffier L, Slatkin M (1995) Maximum-likelihood estimation of molecular haplotype frequencies in a diploid population. Mol Biol Evol 12: 921–927. 7476138 10.1093/oxfordjournals.molbev.a040269

[49] Zaykin DV, Westfall PH, Young SS, Karnoub MA, Wagner MJ, et al (2002) Testing association of statistically inferred haplotypes with discrete and continuous traits in samples of unrelated individuals. Hum Hered 53: 79–91. 12037407 10.1159/000057986

[50] Gauderman WJ MJ (2006) QUANTO 1.1: A computer program for power and sample size calculations for genetic-epidemiology studies. Available: http://hydauscedu/gxe. Accessed: 2011 Aug.

[51] Studies N-NWGoRiA, Chanock SJ, Manolio T, Boehnke M, Boerwinkle E, et al (2007) Replicating genotype-phenotype associations. Nature 447: 655–660. 17554299 10.1038/447655a

