# Supplementary material for: Heterogeneity of the Stearoyl-CoA desaturase-1 (SCD1) Gene and Metabolic Risk Factors in the EPIC-Potsdam Study
Source: PLoS One. 2012 Nov 6;7(11):e48338. doi: 10.1371/journal.pone.0048338 (PMC3491059; doi:10.1371/journal.pone.0048338)
Supplement: Table S1 — Genotype and allelic frequencies of the SCD1 tag-SNPs across the EPIC-Potsdam subcohort and separately for men and women. (PDF) [file pone.0048338.s001.pdf]

Table S1. Genotype and allelic frequencies of the *SCD1* tag-SNPs across the EPIC-Potsdam subcohort and separately for men and women.

SNP	Group	Genotype N (%)			Allele N (%)	
		AA	AB	BB	A	B
**rs1502593**	Subcohort	679 (31.48)	1072 (49.70)	406 (18.82)	2430 (56)	1884(44)
	Men	233 (28.45)	434 (52.99)	152 (18.56)	900 (55)	738 (45)
	Women	446 (33.33)	638 (47.68)	254 (18.98)	1530 (57)	1146 (43)
**rs522951**	Subcohort	607 (28.14)	1095 (50.76)	455 (21.09)	2309 (54)	2005 (46)
	Men	223 (27.23)	441 (53.85)	155 (18.93)	887 (54)	751 (46)
	Women	384 (28.70)	654 (48.88)	300 (22.42)	1422 (53)	1254 (47)
**rs11190480**	Subcohort	1787 (82.85)	357 (16.55)	13 (0.60)	3931 (91)	383 (9)
	Men	681 (83.15)	136 (16.61)	2 (0.24)	1498 (91)	140 (9)
	Women	1106 (82.66)	221 (16.52)	11 (0.82)	2433 (91)	243 (9)
**rs3071**	Subcohort	944 (43.76)	936 (43.39)	277 (12.84)	2824 (65)	1490 (35)
	Men	356 (43.47)	353 (43.10)	110 (13.43)	1065 (65)	573 (35)
	Women	588 (43.95)	583 (43.57)	167 (12.48)	1759 (66)	917 (34)
**rs3793767**	Subcohort	845 (39.17)	998 (46.27)	314 (14.56)	2688 (62)	1626 (38)
	Men	330 (40.29)	384 (46.89)	105 (12.82)	1044 (64)	594 (36)
	Women	515 (38.49)	614 (45.89)	209 (15.62)	1644 (61)	1032 (39)
**rs10883463**	Subcohort	1840 (85.30)	304 (14.09)	13 (0.60)	3984 (92)	330 (8)
	Men	695 (84.86)	120 (14.65)	4 (0.49)	1510 (92)	128 (8)
	Women	1145 (85.58)	184 (13.75)	9 (0.67)	2474 (92)	202 (8)
**rs508384**	Subcohort	1489 (69.03)	610 (28.28)	58 (2.69)	3588 (83)	726 (17)
	Men	561 (68.50)	244 (29.79)	14 (1.71)	1366 (83)	272 (17)
	Women	928 (69.36)	366 (27.35)	44 (3.29)	2222 (83)	454 (17)

A, most frequent allele in each SNP; B, least frequent allele in each SNP (A>B): rs1502593 (C>T), rs522951 (G>C), rs11190480 (A>G), rs3071 (T>G), rs3793767 (T>C), rs10883463 (T>C), rs508384 (C>A).
