# Supplementary material for: Heterogeneity of the Stearoyl-CoA desaturase-1 (SCD1) Gene and Metabolic Risk Factors in the EPIC-Potsdam Study
Source: PLoS One. 2012 Nov 6;7(11):e48338. doi: 10.1371/journal.pone.0048338 (PMC3491059; doi:10.1371/journal.pone.0048338)
Supplement: Table S2 — Association analysis between the 7 SCD1 tag-SNPs and the 8 investigated metabolic traits in the EPIC-Potsdam Study, (mutually) adjusted for known cardiovascular risk factors. (PDF) [file pone.0048338.s002.pdf]

Table S2. Association analysis between the 7 *SCD1* tag-SNPs and the 8 investigated metabolic traits in the EPIC-Potsdam Study, (mutually) adjusted for known cardiovascular risk factors.

	Triglycerides [a, b] (mg/dL)	BMI [c] (kg/m^2)	WC [c] (cm)	HbA1c [d] (%)	GGT [b] (U/L)	ALT [b] (U/L)	Fetuin-A [c] (mg/dL)	hs-CRP [b] (mg/L)
**rs1502593 (n)**								
0 (679)	94.54 (88.67-100.80)	26.09±0.13	85.86±0.34	6.47 (6.42-6.52)	18.57 (17.63-19.55)	19.47 (18.87-20.10)	0.251±0.002	0.76 (0.69-0.83)
1 (1072)	88.95 (84.55-93.58)	26.21±0.11	86.03±0.27	6.49 (6.46-6.53)	18.65 (17.90-19.43)	19.76 (19.27-20.26)	0.249±0.002	0.75(0.69-0.80)
2 (406)	95.39 (88.10-103.29)	25.88±0.17	85.03±0.44	6.44 (6.38-6.51)	18.71 (17.50-20.00)	20.60 (19.77-21.45)	0.257±0.003	0.77 (0.68-0.87)
P_{add}	0.20	0.28	0.15	0.40	0.98	0.10	0.09	0.92
P_{dom}	0.30	0.86	0.79	0.71	0.87	0.18	0.90	0.90
P_{rec}	0.30	0.15	0.06	0.27	0.90	0.04	0.03	0.74
**rs522951 (n)**								
0 (607)	93.70 (87.68-100.14)	26.02±0.14	85.47±0.36	6.46 (6.41-6.51)	18.24 (17.27-19.26)	19.93 (19.28-20.61)	0.254±0.002	0.75 (0.68-0.83)
1 (1095)	91.26 (86.74-96.00)	26.17±0.11	85.86±0.27	6.49 (6.45-6.53)	19.02 (18.26-19.80)	19.89 (19.40-20.39)	0.249±0.002	0.76 (0.71-0.82)
2 (455)	91.16 (84.52-98.33)	26.08±0.16	86.04±0.42	6.47 (6.41-6.53)	18.26 (17.15-19.45)	19.51 (18.78-20.28)	0.252±0.003	0.75 (0.66-0.84)
P_{add}	0.80	0.92	0.92	0.62	0.38	0.67	0.35	0.94
P_{dom}	0.51	0.46	0.31	0.38	0.36	0.70	0.21	0.88
P_{rec}	0.81	0.87	0.50	0.90	0.48	0.37	0.78	0.81
**rs11190480 (n)**								
0 (1787)	91.10 (87.65-94.69)	26.10±0.08	85.82±0.21	6.48 (6.45-6.51)	18.71 (18.13-19.32)	19.99 (19.61-20.38)	0.25±0.001	0.76 (0.71-0.80)
1 (357)	96.28 (88.11-105.21)	26.14±0.19	85.61±0.47	6.45 (6.39-6.52)	18.42 (17.15-19.78)	18.96 (18.16-19.81)	0.25±0.003	0.75 (0.66-0.85)
2 (13)	103.59 (42.75-251.04)	25.76±0.98	85.86±2.47	6.66 (6.31-7.05)	14.40 (9.92-20.93)	20.90 (18.16-19.81)	0.25±0.016	0.65 (0.33-1.28)
P_{add}	0.52	0.45	0.45	0.46	0.37	0.09	0.57	0.91
P_{dom}	0.26	0.89	0.70	0.57	0.53	0.04	0.29	0.85
P_{rec}	0.79	0.72	0.98	0.32	0.18	0.18	0.82	0.67
**rs3071 (n)**								
0 (944)	91.93 (87.06-97.06)	26.21±0.11	86.00±0.29	6.46 (6.41-6.50)	18.96 (18.14-19.81)	19.64 (19.12-20.17)	0.251±0.002	0.79 (0.73-0.85)

1 (936)	92.54 (87.64-97.71)	25.97±0.12	85.59±0.29	6.49 (6.45-6.53)	18.19 (17.41-19.01)	19.83 (19.30-20.37)	0.252±0.002	0.72 (0.67-0.78)
2 (277)	90.07 (81.73-99.27)	26.23±0.21	85.75±0.53	6.50 (6.43-6.58)	19.07 (17.58-20.67	20.43 (19.45-21.46)	0.251±0.003	0.76 (0.65-0.88)
P_{add}	0.89	0.27	0.62	0.38	0.36	0.39	0.97	0.36
P_{dom}	0.99	0.23	0.34	0.17	0.31	0.37	0.82	0.19
P_{rec}	0.66	0.52	0.94	0.50	0.55	0.20	0.96	0.97
**rs3793767 (n)**								
0 (845)	93.56 (88.42-99.01)	26.17±0.12	85.96±0.31	6.46 (6.41-6.50)	18.83 (17.98-19.72)	19.97 (19.41-20.54)	0.252±0.002	0.73 (0.67-0.80)
1 (998)	90.97 (86.33-95.86)	26.02±0.11	85.59±0.28	6.50 (6.46-6.54)	18.85 (18.07-19.67)	19.82 (19.31-20.34)	0.249±0.002	0.78 (0.72-0.84)
2 (314)	90.55 (82.26-99.68)	26.20±0.20	85.96±0.50	6.47 (6.40-6.54)	17.47 (16.19-18.85)	19.43 (18.55-20.36)	0.254±0.003	0.74 (0.65-0.85)
P_{add}	0.74	0.59	0.96	0.42	0.20	0.62	0.31	0.56
P_{dom}	0.44	0.49	0.46	0.26	0.58	0.51	0.51	0.38
P_{rec}	0.74	0.63	0.72	0.79	0.07	0.37	0.28	0.77
**rs10883463 (n)**								
0 (1840)	92.66 (89.21-96.24)	26.07±0.08	85.69±0.21	6.48 (6.45-6.51)	18.38 (17.81-18.96)	19.77 (19.40-20.15)	0.251±0.001	0.76 (0.72-0.80)
1 (304)	85.11 (76.99-94.09)	26.28±0.20	86.22±0.51	6.47 (6.40-6.55)	20.44 (18.92-22.08)	20.12 (19.19-21.09)	0.251±0.003	0.74 (0.65-0.86)
2 (13)	132.82 (88.81-198.6)5	27.56±0.98	89.15±2.48	6.08 (5.79-6.41)	15.23 (10.48-22.14)	20.34 (16.18-25.56)	0.241±0.016	0.53 (0.27-1.04)
P_{add}	0.06	0.21	0.25	0.06	0.03	0.78	0.84	0.57
P_{dom}	0.28	0.22	0.23	0.56	0.02	0.48	0.96	0.67
P_{rec}	0.07	0.14	0.17	0.02	0.29	0.83	0.56	0.30
**rs508384 (n)**								
0 (1489)	91.70 (87.91-95.67)	26.03±0.9	85.67±0.23	6.49 (6.45-6.52)	18.54 (17.90-19.20)	19.99 (19.57-20.42)	0.252±0.001	0.76 (0.71-0.81)
1 (610)	91.14 (85.08-97.64)	26.25±0.14	85.91±0.36	6.46 (6.41-6.51)	18.87 (17.87-19.93)	19.44 (18.80-20.09)	0.249±0.002	0.75 (0.68-0.83)
2 (58)	106.69 (85.97-132.40)	26.58±0.56	87.49±1.17	6.44 (6.28-6.61)	18.55 (15.53-22.16)	19.64 (17.62-21.89)	0.249±0.008	0.65 (0.47-0.90)
P_{add}	0.39	0.26	0.29	0.62	0.86	0.38	0.47	0.65
P_{dom}	0.84	0.14	0.36	0.34	0.61	0.17	0.17	0.68
P_{rec}	0.17	0.31	0.14	0.66	0.96	0.87	0.74	0.36

Each SNP is coded as 0, 1 and 2 according to the number of minor alleles a participant carries. **a**, based on the 615 participants fasting at blood draw; **b**, geometric means and (95% CI); **c**, means and standard error; **d**, inverse and (95% CI); **e** based on 2077 participants due to missing biomarker data; $\mathbf{P}_{\mathbf{add}}$, P for trend or P for the additive model; $\mathbf{P}_{\mathbf{dom}}$, P value for the dominant model; $\mathbf{P}_{\mathbf{rec}}$, P value for the recessive model. All the reported significance levels are nominal P values and are not adjusted for multiple comparisons. All analyses are (mutually) adjusted for age, sex, smoking status (never smoker, former smoker, current smoker <20 cigarettes per day, current smoker ≥20 cigarettes per day), sports activity (<2 h/wk versus ≥2 h/wk), educational attainment (vocational school or less, technical school, university), BMI (continuous), WC (continuous), alcohol consumption (men: = 0 g/d, >0 to 12 g/d, >12 to 24 g/d; >24 g/d; women: = 0 g/d, >0 to 6 g/d, >6 to 12 g/d; >12 g/d), prevalent diabetes, prevalent hypertension, total cholesterol, HDL-cholesterol and hs-CRP.
