# Supplementary material for: Heterogeneity of the Stearoyl-CoA desaturase-1 (SCD1) Gene and Metabolic Risk Factors in the EPIC-Potsdam Study
Source: PLoS One. 2012 Nov 6;7(11):e48338. doi: 10.1371/journal.pone.0048338 (PMC3491059; doi:10.1371/journal.pone.0048338)
Supplement: Table S3 — β regression coefficients for the age- and sex-adjusted association analysis between the SCD1 tag-SNPs and inferred haplotypes and the 8 investigated metabolic traits in the EPIC-Potsdam study. (PDF) [file pone.0048338.s003.pdf]

Table S3. β regression coefficients for the age- and sex-adjusted association analysis between the *SCD1* tag-SNPs and inferred haplotypes and the 8 investigated metabolic traits in the EPIC-Potsdam study.

	N	Ln (Triglycerides) [a] (mg/dL)	BMI (kg/m^2)	WC (cm)	1/(HbA1c) (%)	Ln (GGT) (U/L)	Ln (ALT) (U/L)	Fetuin-A[b] (mg/dL)	Ln (hs-CRP) (mg/L)
**rs1502593**									
β (SE) $_{add}$	0 (679)	-0.004 (0.031)	-0.09 (0.13)	-0.38 (0.33)	0.0002 (0.0005)	0.004 (0.024)	0.02 (0.01)	0.002 (0.002)	-0.01 (0.04)
β (SE) $_{dom}$	1 (1072)	-0.03 (0.05)	-0.04 (0.19)	-0.31 (0.49)	-0.0002 (0.0007)	0.01 (0.04)	0.03 (0.02)	0.0009 (0.003)	-0.01 (0.06)
β (SE) $_{rec}$	2 (406)	0.03 (0.05)	-0.24 (0.23)	-0.78 (0.59)	0.0010 (0.0009)	0.004 (0.042)	0.04 (0.03)	0.006 (0.003)	-0.03 (0.08)
P $_{add}$		0.89	0.47	0.25	0.63	0.87	0.11	0.19	0.88
P $_{dom}$		0.53	0.83	0.53	0.80	0.87	0.24	0.75	0.86
P $_{rec}$		0.63	0.30	0.18	0.25	0.93	0.14	0.06	0.96
**rs522951**									
β (SE) $_{add}$	0 (607)	-0.003 (0.031)	0.14 (0.13)	0.53 (0.33)	-0.0003 (0.0005)	0.003 (0.02)	-0.01(0.01)	-0.001 (0.002)	0.01 (0.04)
β (SE) $_{dom}$	1 (1095)	0.01 (0.05)	0.22 (0.20)	0.65 (0.51)	-0.0007 (0.0008)	0.02 (0.04)	-0.01(0.02)	-0.004 (0.003)	0.02 (0.07)
β (SE) $_{rec}$	2 (455)	-0.02 (0.05)	0.14 (0.22)	0.77 (0.56)	-0.0001 (0.0008)	-0.01 (0.04)	-0.01(0.02)	0.0005 (0.0031)	0.01 (0.07)
P $_{add}$		0.93	0.28	0.10	0.50	0.90	0.70	0.46	0.82
P $_{dom}$		0.90	0.27	0.20	0.35	0.60	0.78	0.20	0.80
P $_{rec}$		0.77	0.52	0.17	0.90	0.72	0.73	0.87	0.91
**rs11190480**									
β (SE) $_{add}$	0 (1787)	0.03 (0.06)	-0.20 (0.23)	-0.66 (0.58)	0.0004 (0.0009)	-0.03 (0.04)	-0.05 (0.02)	-0.003 (0.003)	-0.02 (0.07)
β (SE) $_{dom}$	1 (357)	0.03 (0.06)	-0.19 (0.24)	-0.71 (0.61)	0.0006 (0.0009)	-0.03 (0.04)	-0.06 (0.03)	-0.003 (0.003)	-0.01 (0.08)
β (SE) $_{rec}$	2 (13)	0.20 (0.54)	-0.60 (1.16)	-0.55 (2.96)	0.0039 (0.0044)	-0.27 (0.22)	0.04 (0.13)	-0.006 (0.017)	-0.21 (0.38)
P $_{add}$		0.56	0.39	0.25	0.63	0.41	0.05	0.35	0.78
P $_{dom}$		0.58	0.42	0.24	0.49	0.54	0.03	0.36	0.85
P $_{rec}$		0.72	0.61	0.85	0.38	0.21	0.77	0.73	0.59
**rs3071**									
β (SE) $_{add}$	0 (944)	-0.02 (0.03)	-0.10 (0.13)	-0.30 (0.33)	-0.001 (0.001)	-0.02 (0.02)	0.01 (0.01)	-0.0002 (0.0018)	-0.05 (0.04)
β (SE) $_{dom}$	1 (936)	-0.01 (0.04)	-0.30 (18)	-0.70 (0.46)	-0.001 (0.001)	-0.04 (0.03)	0.01 (0.02)	0.0004 (0.0025)	-0.11 (0.06)
β (SE) $_{rec}$	2 (277)	-0.04 (0.06)	0.26 (0.27)	0.27 (0.68)	-0.001 (0.001)	0.01 (0.05)	0.02 (0.03)	-0.002 (0.004)	0.01 (0.09)
P $_{add}$		0.62	0.47	0.36	0.28	0.46	0.46	0.92	0.22
P $_{dom}$		0.79	0.10	0.13	0.32	0.27	0.62	0.86	0.08
P $_{rec}$		0.54	0.34	0.69	0.46	0.92	0.43	0.64	0.90
**rs3793767**									
β (SE) $_{add}$	0 (845)	-0.03 (0.03)	0.09 (0.13)	0.19 (0.33)	-0.0003 (0.0005)	-0.03 (0.02)	-0.01 (0.01)	0.0001 (0.0018)	0.02 (0.04)
β (SE) $_{dom}$	1 (998)	-0.03 (0.04)	0.04 (0.18)	0.08 (0.47)	-0.0008 (0.0007)	-0.02 (0.03)	-0.01 (0.02)	-0.002 (0.003)	0.04 (0.06)

β (SE) $_{rec}$	2 (314)	-0.06 (0.06)	0.25 (0.26)	0.57 (0.65)	0.0002 (0.0010)	-0.08 (0.05)	-0.02 (0.03)	0.004 (0.004)	-0.003 (0.008)
P $_{add}$		0.37	0.50	0.57	0.50	0.17	0.38	0.97	0.65
P $_{dom}$		0.56	0.81	0.86	0.26	0.49	0.48	0.50	0.50
P $_{rec}$		0.34	0.33	0.38	0.81	0.09	0.47	0.32	0.97
**rs10883463**									
β (SE) $_{add}$	0 (1840)	0.02 (0.06)	0.16 (.24)	0.43 (0.61)	0.0008 (0.0009)	0.09 (0.04)	0.03 (0.03)	-0.001 (0.003)	-0.02(0.08)
β (SE) $_{dom}$	1 (304)	-0.02 (0.06)	0.12 (0.25)	0.32 (0.65)	0.0005 (0.0010)	0.11 (0.05)	0.03 (0.03)	0.00003 (0.004)	-0.01(0.08)
β (SE) $_{rec}$	2 (13)	0.54 (0.24)	1.30 (1.16)	3.54 (2.96)	0.0098 (0.0044)	-0.12 (0.22)	0.07 (0.13)	-0.017 (0.017)	-0.18(0.38)
P $_{add}$		0.76	0.50	0.48	0.36	0.04	0.27	0.85	0.81
P $_{dom}$		0.80	0.64	0.63	0.62	0.02	0.30	0.99	0.88
P $_{rec}$		0.03	0.26	0.23	0.03	0.56	0.56	0.32	0.63
**rs508384**									
β (SE) $_{add}$	0 (1489)	0.04 (0.04)	0.04 (0.17)	0.02 (0.44)	0.0006 (0.0007)	0.03 (0.03)	-0.02 (0.02)	-0.002 (0.002)	-0.01 (0.06)
β (SE) $_{dom}$	1 (610)	0.02 (0.05)	0.05 (0.19)	-0.05 (0.50)	0.0006 (0.0007)	0.03 (0.04)	-0.02 (0.02)	-0.003 (0.003)	0.004 (0.064)
β (SE) $_{rec}$	2 (58)	0.24 (0.13)	-0.01 (0.56)	0.66 (1.42)	0.0012 (0.0021)	0.02 (0.10)	-0.001 (0.061)	-0.005 (0.008)	-0.13 (0.18)
P $_{add}$		0.36	0.81	0.96	0.36	0.40	0.39	0.27	0.86
P $_{dom}$		0.69	0.78	0.92	0.39	0.38	0.33	0.31	0.95
P $_{rec}$		0.07	0.98	0.64	0.58	0.82	0.99	0.49	0.46
**A-B-A-A-B-A-A**									
β (SE) $_{add}$	0 (919)	-0.02 (0.03)	0.06 (0.13)	0.23 (0.34)	-0.0003 (0.0005)	-0.04 (0.03)	-0.01 (0.01)	-0.0007 (0.0019)	0.02 (0.04)
β (SE) $_{dom}$	1 (962)	-0.03 (0.04)	0.04 (0.18)	0.16 (0.47)	-0.0006 (0.0007)	-0.03 (0.03)	-0.02 (0.02)	-0.002 (0.003)	0.05 (0.06)
β (SE) $_{rec}$	2 (250)	-0.03 (0.07)	0.19 (0.28)	0.62 (0.72)	0.0002 (0.0011)	-0.08 (0.05)	-0.02 (0.03)	0.0006 (0.0039)	-0.01 (0.09)
P $_{add}$		0.49	0.63	0.51	0.58	0.15	0.34	0.70	0.59
P $_{dom}$		0.54	0.83	0.74	0.37	0.31	0.44	0.54	0.44
P $_{rec}$		0.62	0.51	0.39	0.82	0.14	0.42	0.89	0.94
**B-A-A-B-A-A-A**									
β (SE) $_{add}$	0 (1041)	-0.02 (0.03)	-0.13 (0.14)	-0.54 (0.35)	-0.0002 (0.0005)	-0.03 (0.03)	0.01 (0.02)	0.0002 (0.0019)	-0.052 (0.045)
β (SE) $_{dom}$	1 (885)	-0.01 (0.04)	-0.21 (0.18)	-0.68 (0.46)	-0.0005 (0.0007)	-0.05 (0.03)	0.01 (0.02)	-0.0006 (0.0025)	-0.08 (0.06)
β (SE) $_{rec}$	2 (201)	-0.09 (0.07)	-0.05 (0.31)	-0.72 (0.79)	-0.0007 (0.0012)	-0.01 (0.06)	0.03 (0.03)	0.0025 (0.0043)	-0.02 (0.10)
P $_{add}$		0.49	0.63	0.51	0.58	0.15	0.34	0.70	0.59
P $_{dom}$		0.54	0.83	0.74	0.37	0.31	0.44	0.54	0.44
P $_{rec}$		0.62	0.51	0.39	0.82	0.14	0.42	0.89	0.94
**B-A-A-A-A-A-A**									
β (SE) $_{add}$	0 (1213)	0.04 (0.05)	-0.06 (0.22)	0.19 (0.56)	0.0008 (0.0008)	0.07 (0.04)	0.02 (0.02)	0.003 (0.003)	0.08 (0.07)
β (SE) $_{dom}$	1 (425)	0.05 (0.06)	-0.02 (0.24)	0.36 (0.60)	0.0003 (0.0009)	0.07 (0.04)	0.02 (0.03)	0.002 (0.003)	0.08 (0.08)
β (SE) $_{rec}$	2 (16)	0.02 (0.22)	-0.88 (1.07)	-3.09 (2.70)	0.012 (0.004)	0.14 (0.20)	0.17 (0.11)	0.02 (0.02)	0.23 (0.34)
P $_{add}$		0.46	0.35	0.13	0.75	0.23	0.42	0.93	0.25
P $_{dom}$		0.80	0.25	0.14	0.44	0.14	0.54	0.82	0.16

P_{rec}		0.23	0.88	0.36	0.55	0.87	0.46	0.56	0.86
**A-A-B-A-A-A-B**									
β (SE) $_{add}$	0 (1795)	0.03 (0.06)	-0.14 (0.23)	-0.55 (0.58)	0.0003 (0.0009)	-0.02 (0.04)	-0.05 (0.03)	-0.003 (0.003)	-0.01 (0.07)
β (SE) $_{dom}$	1 (348)	0.03 (0.06)	-0.15 (0.24)	-0.63 (0.61)	0.0006 (0.0009)	-0.02 (0.04)	-0.06 (0.03)	-0.004 (0.003)	-0.003 (0.078)
β (SE) $_{rec}$	2 (12)	0.20 (0.54)	-0.10 (1.2)	0.63 (3.08)	-0.005 (0.005)	-0.16 (0.22)	0.05 (0.13)	-0.0034 (0.017)	-0.25 (0.40)
P_{add}		0.57	0.54	0.35	0.70	0.59	0.04	0.32	0.88
P_{dom}		0.60	0.52	0.31	0.55	0.67	0.02	0.29	0.97
P_{rec}		0.72	0.94	0.84	0.33	0.47	0.70	0.87	0.53
**A-B-A-A-A-B-B**									
β (SE) $_{add}$	0 (1855)	0.03 (0.06)	0.25 (0.25)	0.66 (0.63)	0.0005 (0.0010)	0.09 (0.05)	0.03 (0.03)	-0.001 (0.003)	-0.02 (0.08)
β (SE) $_{dom}$	1 (275)	-0.003 (0.066)	0.20 (0.26)	0.52 (0.67)	0.00003 (0.0010)	0.11 (0.05)	0.03 (0.03)	-0.0004 (0.0037)	-0.02 (0.09)
β (SE) $_{rec}$	2 (12)	0.65 (0.27)	1.71 (1.21)	4.82 (3.07)	0.010 (0.005)	-0.10 (0.22)	0.10 (0.13)	-0.02 (0.02)	-0.10 (0.39)
P_{add}		0.62	0.32	0.30	0.63	0.05	0.25	0.75	0.76
P_{dom}		0.96	0.45	0.44	0.97	0.03	0.28	0.91	0.79
P_{rec}		0.02	0.16	0.12	0.03	0.64	0.48	0.30	0.80

Each SNP and haplotype is coded as 0, 1 and 2 according to the number of copies of the least common variant a participant carries. Haplotypes are composed of variants rs1502593 (C>T), rs522951 (G>C), rs11190480 (A>G), rs3071 (T>G), rs3793767 (T>C), rs10883463 (T>C), rs508384 (C>A) in that order. A indicates common allele, B indicates rare allele. **β (SE)**, regression coefficient and standard error obtained in linear regression analysis. **a**, based on the 615 participants fasting at blood draw; **b,** based on 2077 participants due to missing biomarker data. All the reported significance levels are nominal P values and are not adjusted for multiple comparisons. **P_{add}**, P for trend or P for the additive model; **P_{dom}**, P value for the dominant model. **P_{rec}**, P value for the recessive model.
